# Hysteroscopic treatment of complete uterine septum, double cervix and longitudinal vaginal septum (U2bC2V1): the use of a Foley catheter balloon

**DOI:** 10.52054/FVVO.16.4.042

**Published:** 2024-12-27

**Authors:** O Triantafyllidou, E.K. Panagodimou, N Syggelos, N.F. Vlahos

**Affiliations:** 2nd Obstetrics and Gynecology Clinic of University of Athens, Aretaieio Hospital, 11528 Athens, Greece

**Keywords:** Hysteroscopy, Müllerian anomalies, U2bC2V1, metroplasty, uterine septum

## Abstract

This is the case of a 30-year-old nulliparous patient with a complete uterine septum, double cervix and non- obstructive longitudinal vaginal septum (Class U2bC2V1 according to the ESHRE/ESGE classification). The patient presented with severe dyspareunia and dysmenorrhea. Imaging revealed a complex Müllerian anomaly and hysteroscopic treatment was agreed. We present an approach of hysteroscopic metroplasty after insertion of a Foley catheter balloon in one uterine hemi-cavity, which serves as a guide for septum resection using a resectoscope in the contralateral hemi-cavity. No complications were encountered. The patient was discharged after a short period of observation. A post-operative evaluation revealed complete resection of the vaginal septum and the formation of a single, normal uterine cavity.

## Introduction

Congenital uterine anomalies (CUAs) refer to anatomic anomalies of the female genital tract due to abnormal fusion or resorption of the Müllerian (aka paramesonephric) ducts. The incidence of these anomalies is estimated to range from 0.001% to 10% in the general population (Louden et al., 2015). Septate uterus is the most common Müllerian anomaly (Valle and Ekpo, 2013; Blanco-Breindel et al., 2023). A complete uterine septum with double cervix and non-obstructive longitudinal vaginal septum is classified as U2bC2V1 according to the European Society of Human Reproduction and Embryology / European Society for Gynaecological Endoscopy (ESHRE/ESGE) system. (Grimbizis et al., 2013). This represents a complex congenital anomaly of the genital tract. Patients may present with dyspareunia, infertility, recurrent miscarriages or preterm labour (Chan et al., 2011).

The exact prevalence of U2bC2V1 anomaly cannot be estimated, as it is rare and not systematically reported. In the past, it has been frequently misdiagnosed, but the increasing use of 3D ultrasound and magnetic resonance has led to more accurate and prompt diagnosis (Di Spiezio Sardo et al., 2016; Udayakumar et al., 2023). The appropriate management of these patients may not always be clear, as the literature mostly consists of case reports (Pozzati et al., 2023) and retrospective case series (Di Spiezio Sardo et al., 2021; Zizolfi et al., 2023).

Hysteroscopic treatment is considered the gold standard surgical intervention for these patients. A longitudinal vaginal septum can be resected in symptomatic patients, with various described techniques (Grynberg et al., 2012), while there is no indication for surgical treatment of a “normal” duplicated cervix (Ludwin et al., 2013). Hysteroscopic uterine septum resection is associated with increased live birth rates and reduced miscarriage rates (Jiang et al., 2023) or reduced rates of spontaneous abortions (Noventa et al., 2022). Hysteroscopic metroplasty is associated with improved fertility and obstetric outcomes (Parodi et al., 2022).

Hysteroscopy in the context of this complex anomaly can prove demanding for the surgeon. In such cases, the most challenging part of the procedure is to create the initial incision at the lower part of the uterine septum and enter the contralateral hemi-cavity. Currently, the surgical technique lacks standardisation and relies primarily on expert opinion (Di Spiezio Sardo et al., 2021). In this case report, we propose a technique for initial septum incision using a Foley catheter balloon in the contralateral hemi-cavity as a guide.

## Patient and method

**Figure 1:**
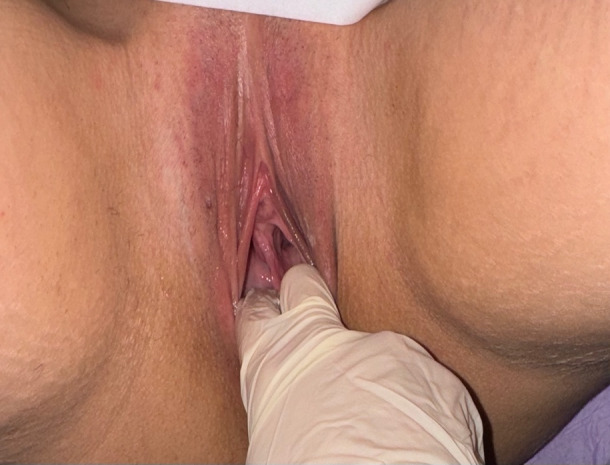
Longitudinal vaginal septum during physical examination.

**Figure 2:**
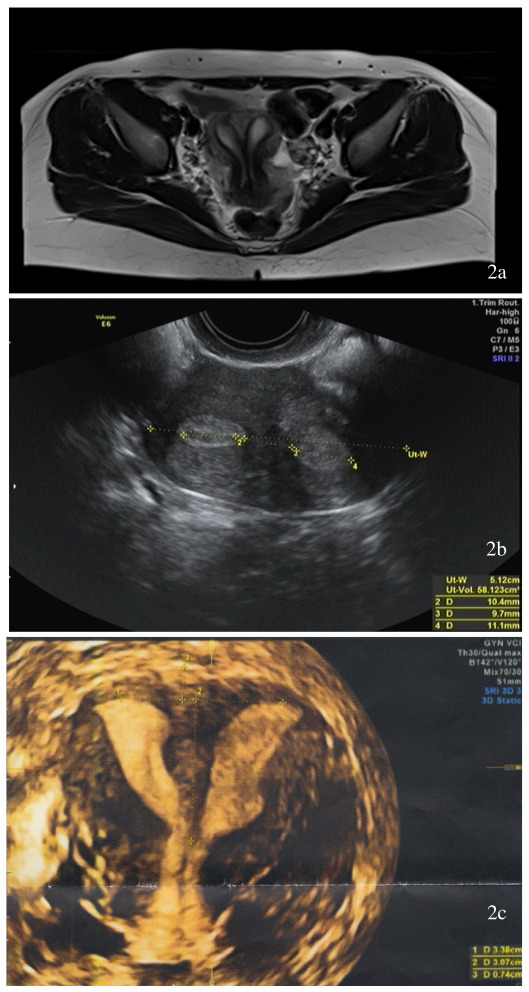
(a) MRI findings before the hysteroscopic resection: complete septate uterus with double “normal” cervix and longitudinal non-obstructing vaginal septum. (b) 2D transvaginal ultrasound. (c) 3D transvaginal ultrasound.

A 30-year-old nulliparous patient presented to our department for consultation due to severe dyspareunia and dysmenorrhoea. The gynaecological examination revealed a longitudinal non-obstructing vaginal septum (Figure 1) with a well-formed cervix on the right side and a hypoplastic cervix on the left side. During 2D ultrasound, the presence of a uterine septum was suspected in a transverse scan. Subsequent magnetic resonance imaging revealed a convex external contour of the uterus, the presence of a complete uterine septum, two uterine hemi- cavities and two cervices. This was identified to be a U2bC2V1 malformation, according to the ESHRE/ ESGE classification (Figure 2a-c). Hysteroscopy was decided as the next step of management, after obtaining the patient’s informed written consent.

Hysteroscopy was scheduled in the early proliferative phase of the menstrual cycle and performed under general anaesthesia. Initially, the longitudinal vaginal septum was resected from the anterior and posterior vaginal wall using a monopolar knife electrode, until the double cervix was exposed. Then, diagnostic hysteroscopy was performed using a 4mm hysteroscope (B.I.O.H. or “Bettocchi Size 4”, Karl Storz Co, Tuttlingen, Germany) with a stable intrauterine pressure at 45 mm/Hg maintained by a fluid management pump (Hysteromat E.A.S.I., Karl Storz Co, Tuttlingen, Germany). Complete vaginal septum excision was confirmed and the hysteroscope was inserted through each cervical orifice, without encountering cervical stenosis. Two non-communicating uterine hemi-cavities were recognized, each with a normal tubal ostium.

Thereafter, a No 16 Foley balloon catheter was inserted through the left cervix in the left uterine hemi-cavity. The tip of the catheter was removed with scissors just above the balloon (so that the balloon now forms the tip of the catheter). Then, the catheter was grasped by a Bonney uterine polyp forceps (with narrow jaws and 6mm tip width) and was gradually inserted into the left uterine hemi- cavity. The absence of cervical stenosis and the use of grasping forceps facilitated the smooth insertion of the Foley catheter, which was subsequently inflated with 6 ml of saline to maintain its position. Correct placement and distention of the left hemi- cavity were verified by transabdominal ultrasound.

**Figure 3:**
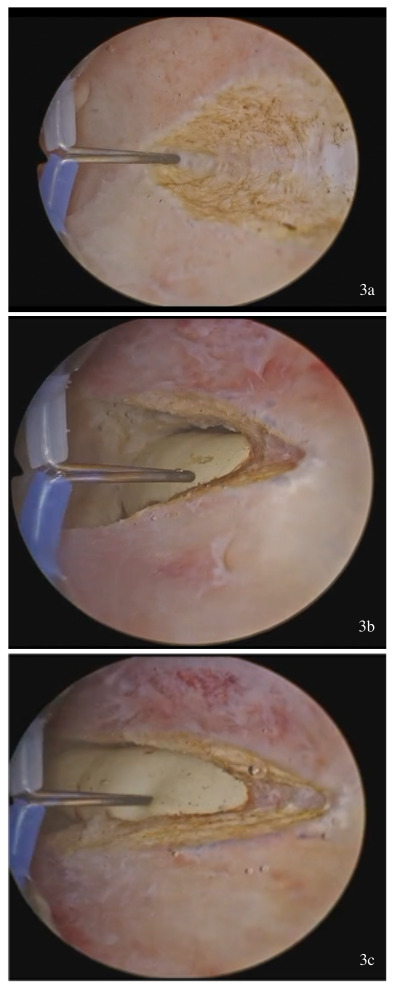
(a-c) The initial incision of the uterine septum at the point of maximum projection of the foley catheter balloon in the contralateral semicavity. The foley catheter balloon in the contralateral semicavity, serving as a guiding point for septum resection.

**Figure 4:**
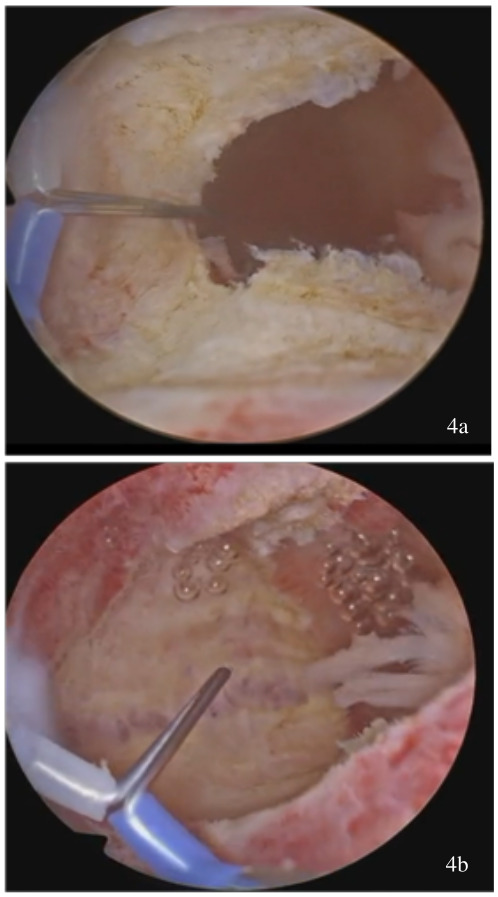
(a-b) Further extension of the incision.

**Figure 5:**
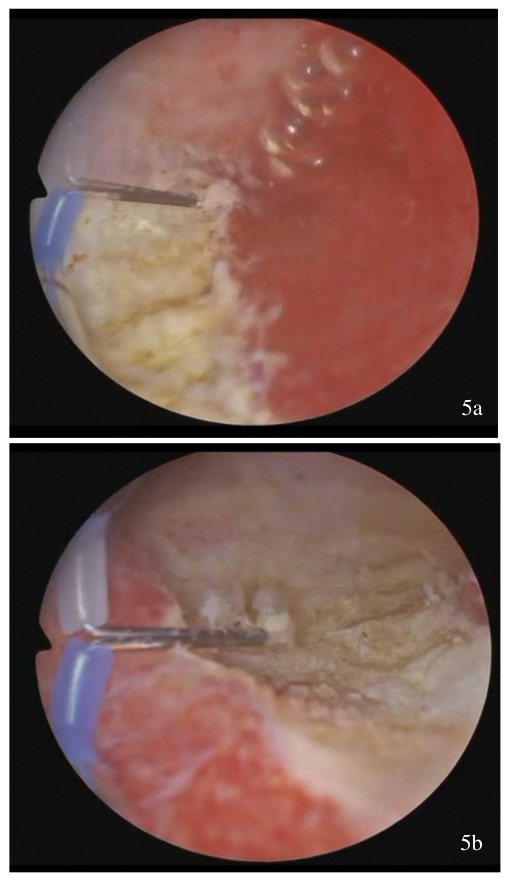
(a-b) Resection of redundant tissue at the level of the anterior and posterior uterine wall.

Following right cervical dilation with Hegar dilators up to size 9, an 8.5 mm continuous flow hystero-resectoscope (Olympus Co, Hamburg, Germany) was inserted in the right uterine hemi- cavity, intrauterine pressure maintained by a fluid management pump between 80 to 100 mm Hg. The performed technique was as follows:

1. incision of the uterine septum with a bipolar electrode hook at the point of its maximum projection near the isthmic level (Figure 3a)
2. the balloon is identified and communication of the two hemi-cavities is achieved (Figure 3b)
3. the balloon serves as a landmark, over which the electrode “slides” (Figure 3c) and extends the initial incision of the septum from the apex to the base, reaching the interostial plane. A single uterine cavity containing the catheter balloon is recognized (Figure 4a, b)
4. removal of the catheter and resection of redundant tissue at the level of the anterior and posterior uterine wall (Fascilla et al., 2020) using the same instrument (Figure 5a, b)

## Results

**Figure 6:**
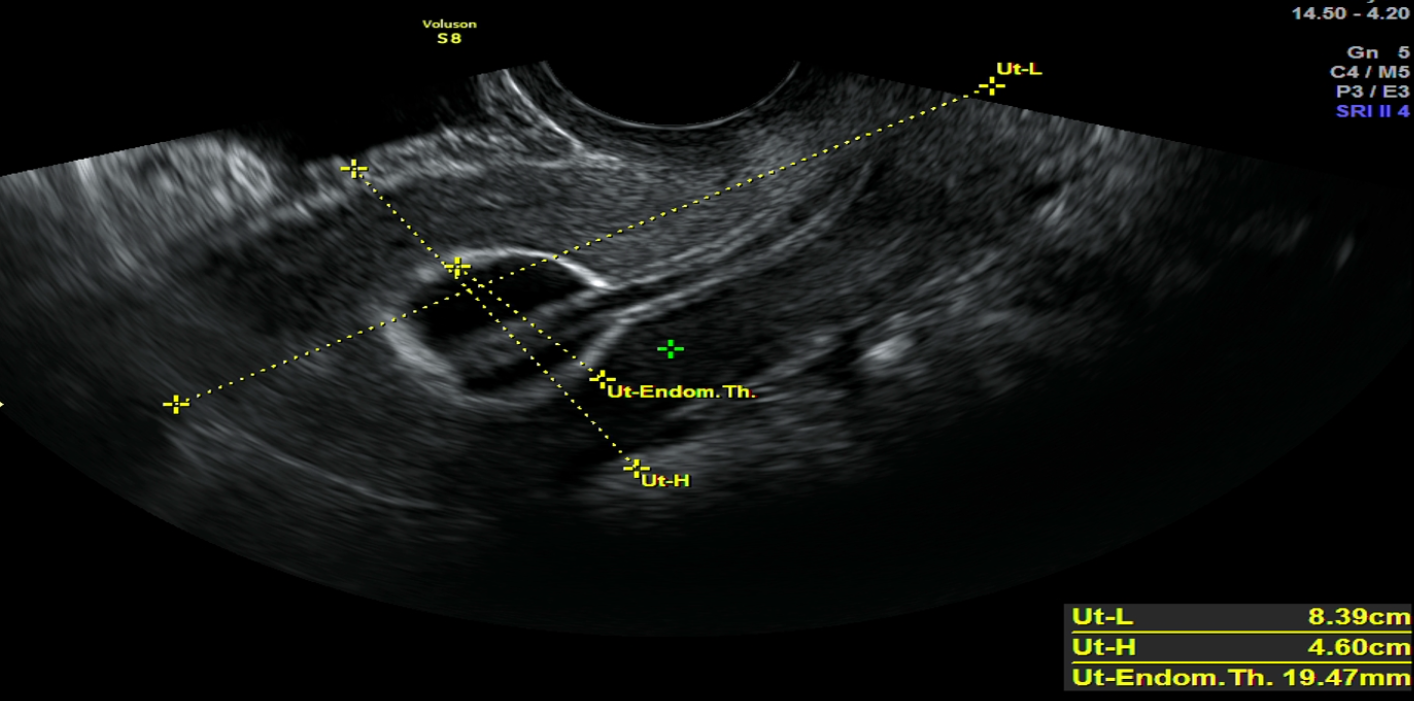
2D ultrasound image. A foley catheter balloon filled with 6 ml of normal saline in the reconstructed uterine cavity.

**Figure 7:**
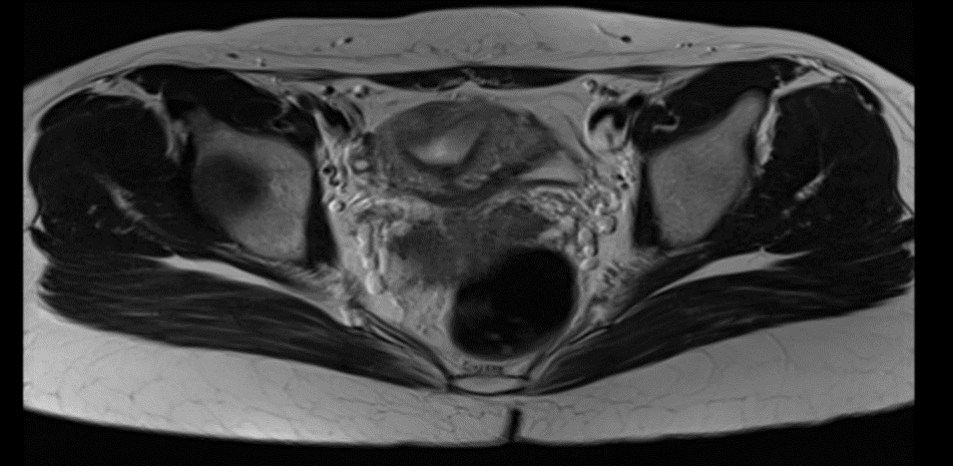
MRI findings two months post hysteroscopic metroplasty confirm complete septum resection and a normal uterine cavity.

The procedure was completed with insertion of a new Foley catheter into the reconstructed uterine cavity, which was filled with 6ml of normal saline solution. The patient was discharged the same day after a short period of observation, without any complications. The final catheter remained for 10 days postoperatively, in order to minimise intrauterine adhesion formation (Figure 6). The patient received oral doxycycline (100mg twice daily for 10 days), oral valerate oestradiol (2mg thrice daily for 20 days) and levonorgestrel (0.5mg daily for the following 10 days) postoperatively. The patient was re-examined after a 6-week interval, in an office setting, clinical examination with a small speculum confirmed a complete resection of the vaginal septum and transvaginal 2D ultrasound identified a normal external uterine outline and a single uterine cavity. Follow-up pelvic MRI two months after the procedure confirmed complete septum resection (Figure 7).

## Discussion

While women with a septate uterus can carry pregnancies to term (Rikken et al., 2021), hysteroscopic metroplasty has been associated with improved fertility and obstetric outcomes (Zhang et al., 2022; Carrera et al., 2022; Jiang et al., 2023). There is a lack of consensus that a Class U2bC2V1 anomaly (Grimbizis et al., 2013) should be surgically treated. Hysteroscopic septum resection has proved beneficial for symptomatic patients (Noventa et al., 2022; Parodi et al., 2022) and should be offered to selected patients.

Initial incision of a complete uterine septum remains the part of the procedure with the highest inter-operator variability in these patients. The thinnest part of the septum may be indicated by preoperative MRI (Chen et al., 2023), a light- source (Yang et al., 2014) or a Hegar dilator (Di Spiezio Sardo et al., 2021) in the contralateral hemi-cavity or concurrent use of ultrasound (Pozzati et al., 2023; Blanch Fons et al., 2020). In this case, we propose the use of a Foley catheter balloon as a guide in the non-working hemi-cavity. The balloon illustrates the best site for septum incision at the point of maximum projection. This can be individualised according to cavity size by inflation of the balloon to the volume desired by the surgeon. Such dynamic manipulation is not possible with fixed size instruments, like Hegar dilators (Di Spiezio Sardo et al., 2021; Zizolfi et al., 2023). The balloon also serves as a means of protection from injury of the uterine walls, as it completely occupies one hemi-cavity, and facilitates visualisation by preventing loss of distention medium from one cervix.

3D ultrasound and miniaturised instruments are key elements for successful septum resection in expert centres (Di Spiezio Sardo et al., 2023). The main advantage of this technique is that it is simple, safe and low-cost making it replicable in less equipped settings. We consider it feasible with any operative hysteroscope or mini-resectoscope. There is no need for preoperative office hysteroscopy as diagnostic hysteroscopy is integrated in the first part of the procedure. The Foley catheter eliminates the need for ultrasound guidance, which can be limited by intestinal meteorism, elevated BMI and a retroverted uterus (Pozzati et al., 2023). The only issue that may arise is difficulty in inserting the catheter through a stenosed cervix.

The main disadvantage of this paper is that it is a single case presentation, though we intend to integrate the technique into our practice for similar cases in the future. The other serious limitation of our approach is that re-look hysteroscopy was not performed. This would be the ideal means of postoperative assessment of the uterine cavity (and simultaneous treatment in case of residual septum or adhesions). A speculum examination verified complete vaginal septum resection. The patient declined re-look hysteroscopy and was counselled that this would be advisable before attempting conception. Instead, we performed a follow-up MRI for postoperative evaluation, which confirmed successful treatment.

## Conclusion

Hysteroscopic metroplasty can prove challenging in patients with a complete uterine septum, double cervix and longitudinal vaginal septum (Class U2bC2V1 Müllerian malformation). A Foley catheter balloon can be used as a guide in the non- operative hemi-cavity. The balloon illustrates the best site for septum incision and reduces the risk of complications. The procedure was concluded with minimal operative time, steps and cost. Follow-up MRI confirmed complete septum resection and normal uterine cavity formation, though second-look hysteroscopy was not performed. Our experience with this technique yielded very promising results and we consider it an opportunity for further investigation.
